# Analysis of 29 Items of the Meaningful Activity Participation Assessment-Meaningful Scale Through Rasch Analysis

**DOI:** 10.1093/geroni/igab046.2075

**Published:** 2021-12-17

**Authors:** Sanghun Nam, Suyeong Bae, Ickpyo Hong

**Affiliations:** Yonsei University, Wonju, Kangwon-do, Republic of Korea

## Abstract

Individuals find meaning in their personal activities. Meaningful activities can improve an individual's emotional and physical health and quality of life. The Meaningful Activity Participation Assessment-Meaningful Scale (MAPA-M), which can measure these meaningful activities, is measured in 29 items. In this study, the psychometric properties of 29 items of MAPA-M were investigated through Rasch analysis. The data used in this study was the Well Elderly Study 2 data among public data provided by the Inter-university Consortium for Political and Social Research (ICPSR). We used 480 randomized samples from the Well Elderly Study 2 data. Before proceeding with the Rasch analysis, as a result of checking the unidimensionality assumption of 29 items, 19 items satisfied the unidimensionality assumption. As a result of Rasch analysis of 19 items, the Driving item was removed as misfit (infit mean-square = 2.04, infit z-standardized fit statistics = 9.90, outfit mean-square = 1.86, outfit z-standardized fit statistics = 8.99). The 18 items with the misfit items removed show a conceptual item-difficulty hierarchy, and there was no differential item functioning that worked for sex and age groups. The person strata value is 3.97, which corresponds to the confidence value of 0.88. These results indicate that the 18 items in MAPA-M show appropriate item-level psychometric properties. In other words, the modified MAPA-M 18 indicates that meaningful activities can be accurately and stably measured.

